# Patients with first-episode, drug-naive schizophrenia and subjects at ultra-high risk of psychosis shared increased cerebellar-default mode network connectivity at rest

**DOI:** 10.1038/srep26124

**Published:** 2016-05-18

**Authors:** Houliang Wang, Wenbin Guo, Feng Liu, Guodong Wang, Hailong Lyu, Renrong Wu, Jindong Chen, Shuai Wang, Lehua Li, Jingping Zhao

**Affiliations:** 1 Mental Health Institute of the Second Xiangya Hospital, Central South University, The China National Clinical Research Center for Mental Health Disorders, National Technology Institute of Psychiatry, Key Laboratory of Psychiatry and Mental Health of Hunan Province, 139 Middle Renmin Road, Changsha, Hunan 410011, People’s Republic of China; 2 Key Laboratory for NeuroInformation of Ministry of Education, School of Life Science and Technology, University of Electronic Science and Technology of China, Chengdu, Sichuan, China

## Abstract

Increased cerebellar-default mode network (DMN) connectivity has been observed in first-episode, drug-naive patients with schizophrenia. However, it remains unclear whether increased cerebellar-DMN connectivity starts earlier than disease onset. Thirty-four ultra-high risk (UHR) subjects, 31 first-episode, drug-naive patients with schizophrenia and 37 healthy controls were enrolled for a resting-state scan. The imaging data were analyzed using the seed-based functional connectivity (FC) method. Compared with the controls, UHR subjects and patients with schizophrenia shared increased connectivity between the right Crus I and bilateral posterior cingulate cortex/precuneus and between Lobule IX and the left superior medial prefrontal cortex. There are positive correlations between the right Crus I-bilateral precuneus connectivity and clinical variables (Structured Interview for Prodromal Syndromes/Positive and Negative Symptom Scale negative symptoms/total scores) in the UHR subjects. Increased cerebellar-DMN connectivity shared by the UHR subjects and the patients not only highlights the importance of the DMN in the pathophysiology of psychosis but also may be a trait alteration for psychosis.

Schizophrenia is a complex and lifelong psychiatric disorder that is conceptualized as a result of aberrant brain connectivity^1^. The “disconnection” hypothesis proposed that disruptive integration of extensive brain regions might account for the underlying pathophysiology of schizophrenia. These brain regions comprise multiple brain networks, such as the cortico-cerebellar-thalamo-cortical network^2^,^3^ and default-mode network (DMN)^4^.

The DMN is one of the most assessed networks in schizophrenia. Abnormal DMN connectivity has been observed in patients with schizophrenia: increased connectivity^4^,^5^,^6^,^7^,^8^, decreased connectivity^9^,^10^,^11^, or both^12^,^13^. Previously, we observed abnormal DMN homogeneity in a group of first-episode, drug-naive patients^14^. The above-mentioned findings highlight the importance of the DMN in the pathophysiology of schizophrenia.

However, it remains unclear that the DMN abnormalities are related to the disease or disease process. One possible solution to this puzzle is to investigate whether these abnormalities are present before disease onset. Recruiting subjects at the ultra-high risk (UHR) phase of psychosis can help to disentangle this puzzle. UHR subjects are adolescents and young adults with imminent risk to develop psychosis^15^. During this phase, UHR subjects may experience some mild psychotic symptoms and cognitive deficits, and seek help from hospital^16^. UHR subjects are estimated to convert to frank psychosis with an average rate of 22% after one year and about 36% after three years^17^. Resting-state functional MRI (fMRI) has revealed that subjects with a familial risk for psychosis have abnormal DMN connectivity between the prefrontal cortex, posterior cingulate cortex (PCC) and precuneus^18^,^19^. A failure to regulate the ventromedial prefrontal cortex and precuneus has been reported in subjects with a familial risk for psychosis during self-referential processes^18^. Studies with UHR subjects observed decreased connectivity between frontal and subcortical regions at rest^20^. Hence, findings from UHR subjects will certainly be applied for early detection and possibly for early intervention^15^.

Human cerebellum has been recognized to participate in the high-order brain function. For example, cognitive deficits have been observed in patients with cerebellar damage^21^,^22^. The cerebellum has functional connectivity (FC) with brain networks, such as the cerebellum Crus I and Lobule IX linking with the DMN^23^,^24^,^25^,^26^,^27^,^28^,^29^. Patients with schizophrenia have cerebellar alterations, such as reduced cerebellar size^30^,^31^ and metabolism^32^,^33^. Decreased FC with the DMN has been found in a group of chronic, medicated patients with schizophrenia^34^. Recently, we observed increased cerebellar-DMN connectivity in first-episode, drug-naive patients with schizophrenia and their unaffected siblings^35^. Although abnormal cerebellar-DMN connectivity has been documented in schizophrenia, the findings are inconsistent^34^,^35^. Hence, conclusions could not be made whether abnormal cerebellar-DMN connectivity is an inherent characteristic to schizophrenia, or it only reflects a state-dependent change. Recruiting UHR subjects who are in the prodromal phase of psychosis might be a good choice to solve this puzzle.

In the present study, UHR subjects and first-episode, drug-naive patients with schizophrenia were recruited to assess the cerebellar-DMN connectivity. The purpose of this study was to explore that abnormal cerebellar-DMN connectivity was a trait alteration (shared by UHR subjects and patients) or a state-dependent process. Based on our previous findings from patients with schizophrenia and their siblings^35^, we hypothesized that UHR subjects and patients with schizophrenia would share increased cerebellar-DMN connectivity. We also examined the correlations between abnormal connectivity and symptom severity in the patients and the UHR subjects.

## Methods and Materials

### Participants

The present study was performed in accordance with the Helsinki Declaration^36^. Thirty-seven UHR subjects and 35 first-episode, drug-naive patients with schizophrenia were recruited from the Mental Health Institute of the Second Xiangya Hospital, Central South University, China, and 40 healthy controls were recruited from the local community. The structured interview for prodromal syndromes (SIPS) and scale of prodromal syndromes (SOPS)^37^ were applied to define the UHR criteria: 1) brief intermittent psychotic syndrome, 2) attenuated positive symptom syndrome, and 3) genetic risk and deterioration syndrome. The SIPS (19 items) assesses four symptom clusters: positive symptoms, negative symptoms, disorganized symptoms, and general symptoms. The SOPS is used to determine the presence of a psychotic syndrome that is either 1) disorganizing or dangerous or 2) occurring at least an hour a day on average four days a week for at least one month. The SIPS/SOPS has exhibited acceptable reliability and validity^37^,^38^. Patients with schizophrenia were diagnosed with the Structured Clinical Interview of the Diagnostic and Statistical Manual of Mental Disorders-IV (SCID) criteria, patient edition^39^. Positive and Negative Symptom Scale (PANSS) was used to determine the symptom severity for the patients. Healthy controls were screened by SCID, non-patient edition^39^. All participants were right-handed and aged from 14 to 30 years old. They had more than 3 years of normal education, and experienced no previous psychotic episodes or treatment with neuroleptics. Exclusion criteria for all participants were neurological disorders, severe medical disorders, mental retardation, substance abuse, or any contraindications for MRI. Healthy controls having a first-degree relative with a psychiatric disorder were also excluded.

The study was approved by the local ethics committee of the Second Xiangya Hospital. All participants gave written informed consent.

### MRI acquisition and Image preprocessing

A Siemens 3T scanner was used to acquire functional MRI images, which was preprocessed with Data Processing Assistant for Resting-State fMRI^40^. Details of MRI acquisition and Image preprocessing can be found in the supplementary files.

### FC analysis

Three seed ROIs were used in FC analysis, including the right Crus I (MNI: 33, −76, −34), left Crus I (MNI: −33, −76, −34), and lobule IX (MNI: 0, −55, −49). These seeds showed intrinsic connectivity with the DMN in healthy subjects^23^,^24^ and patients with schizophrenia^34^,^35^. The ROIs were defined as 6mm radius spheres with the software REST^41^ for FC analysis. Pearson correlation coefficients between each seed and other voxels of the entire brain were computed to create correlation maps that were *z*-transformed with Fisher’s *r*-to-*z* transformation. Details of FC analysis with the software REST can be found in the supplementary files.

For each seed and each group, one-sample *t*-tests were used to detect voxels that showed significant correlations with the seeds. The significance level was set at *p* < 0.005 corrected for multiple comparisons using the Gaussian Random Field (GRF) theory (min *z* > 2.807, cluster significance: *p* < 0.005). GRF correction is a cluster-extent based thresholding, which identifies statistically significant clusters according to the number of contiguous voxels, and controls the estimated false positive probability of the brain region as a whole^42^,^43^. Analyses of covariance (ANCOVA), followed by post hoc *t*-tests, were conducted to compare group differences within the union mask of one-sample *t*-test results of three groups. Age was used as a covariate to minimize the possible effect of this variable. Framewise displacement (FD) for each participant was calculated^44^ and also used as a covariate in the group comparisons. The significance level was set at *p* < 0.005 (GRF corrected).

### Correlation analysis

The mean *z* values of brain regions with abnormal FCs were extracted to examine the correlations between abnormal FCs and clinical variables in the patients and UHR subjects. Multiple linear regressions were performed with abnormal FCs as dependent variables and clinical variables as independent variables in the patients and the UHR subjects. The correlation results were Bonferroni corrected at *p* < 0.05.

## Results

### Sample characteristics

Three UHR subjects, 4 patients with schizophrenia and 3 healthy controls were excluded due to excessive head motion. As shown in Table 1, the three groups have no significant differences in age, sex ratio, education level, and FD values. As expected, the patients got higher PANSS scores than the UHR subjects and healthy controls.

### FC patterns of the seed

For each group, each cerebellar seed (Figure S1) showed distributed FCs with the DMN using one-sample *t*-tests (Figure S2). A union mask for each seed was created based on the results of one-sample *t*-tests for the following seed-based FC analyses.

### Seed-based FCs: Group differences

**Figure 1:**
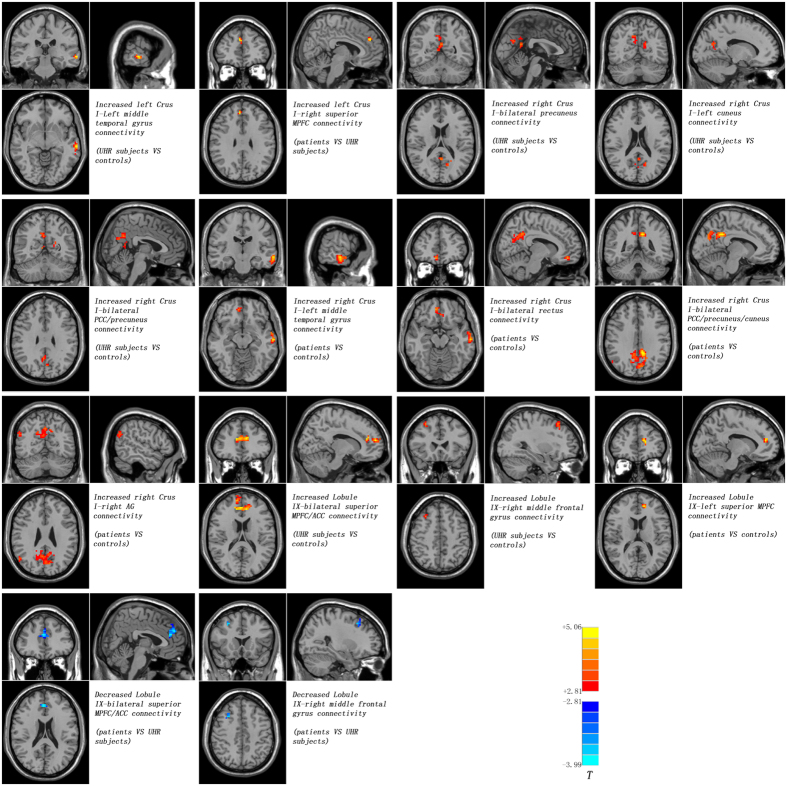
Abnormal cerebellar - DMN connectivity between groups. Red and blue denote increased and decrease FC values. Color bar indicates post-hoc *t* values. DMN = default mode network, MPFC = medial prefrontal cortex; PCC = posterior cingulate cortex; AG = angular gyrus; ACC = anterior cingulate cortex.

Compared with the controls, the UHR subjects exhibited increased FCs between the left Crus I and the left middle temporal gyrus (MTG), between the right Crus I and the left cuneus, bilateral PCC/precuneus and bilateral precuneus, and between the Lobule IX and the right middle frontal cortex and bilateral medial prefrontal cortex (MPFC)/anterior cingulate cortex (ACC) (Fig. 1 and Table 2). By contrast, the patients showed increased FCs between the right Crus I and the left MTG, bilateral rectus, bilateral PCC/precuneus/cuneus and right angular gyrus (AG), and between the Lobule IX and the left superior MPFC relative to the controls (Fig. 1 and Table 2). Compared with the UHR subjects, the patients showed increased FCs between the right Crus I and the right superior MPFC, and decreased FCs between the Lobule IX and the right middle frontal cortex and bilateral superior MPFC/ACC (Fig. 1 and Table 2). Hence, the UHR subjects and the patients shared increased FCs between the right Crus I and bilateral PCC/precuneus, and between the Lobule IX and the left superior MPFC.

### Correlations between abnormal FCs and clinical variables

**Figure 2:**
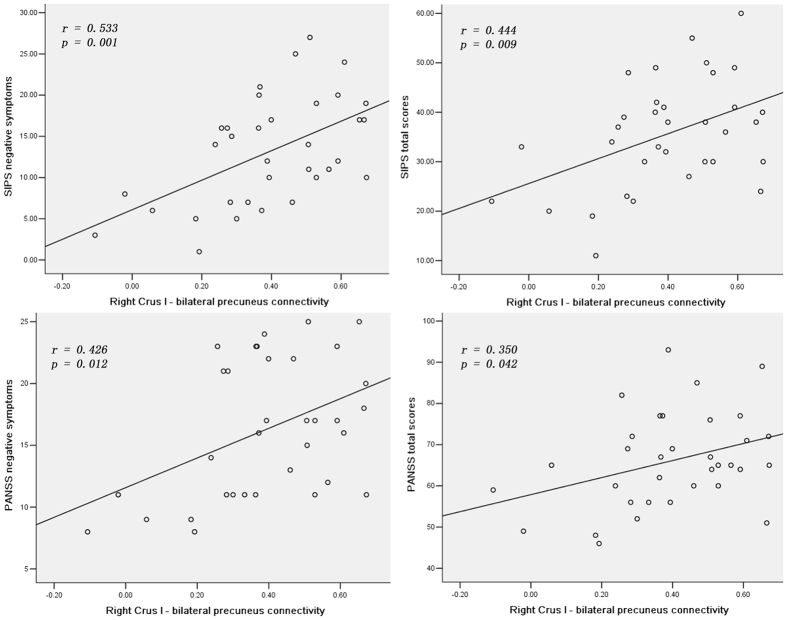
Significantly positive correlations between the mean FC values of the right Crus I - bilateral precuneus connectivity and SIPS/PANSS negative symptoms/total scores in the UHR subjects. SIPS = structured interview for prodromal syndromes; PANSS = Positive and Negative Symptom Scale, FC = functional connectivity, UHR = ultra-high risk.

In the UHR subjects, the mean *z* values of the right Crus I-bilateral precuneus connectivity were positively correlated with the SIPS negative symptoms scores (*r* = 0.533, *p* = 0.001) and total scores (*r* = 0.444, *p* = 0.009), and the PANSS negative symptoms scores (*r* = 0.426, *p* = 0.012) and total scores (*r* = 0.350, *p* = 0.042) (Fig. 2). No correlations were observed between abnormal FCs and clinical variables in the patients. There were also no correlations between abnormal FCs and age, educational level in the patients and UHR subjects.

## Discussion

In the present study, the cerebellar seeds linking with the DMN were used to examine the cerebellar FC patterns with the DMN in the UHR subjects and first-episode, drug-naive patients with schizophrenia. The findings revealed that the UHR subjects and the patients showed increased cerebellar-DMN FCs relative to the controls. UHR subjects and patients with schizophrenia shared increased connectivity between the right Crus I and bilateral PCC/precuneus and between Lobule IX and the left superior MPFC. In addition, positive correlations were found between the cerebellar-DMN connectivity and clinical variables (SIPS/PANSS negative symptoms/total scores) in the UHR subjects.

Previously, we observed that first-episode, drug-naive patients with schizophrenia and their unaffected siblings shared increased cerebellar-DMN connectivity that could be applied as candidate endophenotypes for schizophrenia^35^. Consistent with the previous study and our hypothesis, patients with schizophrenia and UHR subjects shared increased cerebellar-DMN connectivity in this study. Combined with our previous study^35^, the presence of increased cerebellar-DMN connectivity in schizophrenia was advanced from the first-episode stage to the prodromal stage.

However, the present findings are not consistent with the “disconnection” hypothesis in schizophrenia^1^, which hypothesized that schizophrenia have overall decreased connectivity^45^. In support to the “disconnection” hypothesis, Wang *et al.*^34^ found significantly reduced FC between cerebellar seeds and brain regions of the DMN, such as the middle frontal gyrus, ACC, thalamus and supplementary motor area in a group of chronic and medicated patients with schizophrenia. The inconsistency may come from sample heterogeneity in addition to sample size and scanners. The prevailing opinion of overall decreased connectivity in schizophrenia is based on findings with chronic and/or medicated patients. When drug-naive and early-course patients were enrolled, some studies reported increased connectivity^46^,^47^. Moreover, early-course patients exhibited enhanced glutamate concentrations in the frontal gyrus^48^,^49^, which would result in frontal hyperconnectivity. Combined with the findings from early-course and chronic patients, we can depict that the cerebellar-DMN connectivity decreases progressively with illness duration, with increased connectivity in early-course patients and decreased connectivity in chronic patients. Since UHR subjects were enrolled in the present study, the presence of increased connectivity is advanced to the prodromal stage. Compared with the UHR subjects, some cerebellar-DMN connectivities in patients with schizophrenia begin to decrease in this study. By contrast, we previously observed decreased connectivity between the left Crus I and bilateral ACC in another group of first-episode patients^35^. Therefore, we can deduce that cerebellar-DMN connectivity decreases at the early-course of the disease. However, the exact timepoint of the reduction remains unclear. The different findings between two groups of patients may come from different illness duration in addition to different scanners. The mean illness duration of the present patient group is 5.16 months, and the mean illness duration of our previous patient group is 22.45 months. The difference of illness duration between the two studies gives additional support to our speculation that the cerebellar-DMN connectivity decreases progressively with illness duration.

There are two possible interpretations to increased cerebellar-DMN connectivity in UHR subjects and patients with schizophrenia. First, FC differences of the prefrontal-thalamic-cerebellar circuit were compared among healthy children, adolescents and adults with a seed-based FC analysis^50^, and the findings revealed that FC of this circuit exhibits an inverted *U*-curve with maximal point in adolescents. The UHR subjects and the patients in this study are aged from 14 to 30 years, the developmental period from adolescents to adults. In addition, the pathophysiological process of the disease is speculated to start before the disease onset^35^,^51^. Normal developmental process of the cerebellar-DMN connectivity may be halted by the disease in the UHR subjects and the patients, and remain at relatively high point of the inverted *U*-curve. Hence, it is not surprising that the UHR subjects and the patients exhibit increased cerebellar-DMN connectivity in this study. The shared increased cerebellar-DMN connectivity in the UHR subjects and the patients may be a trait alteration for psychosis.

Second, increased cerebellar-DMN connectivity is also meaningful from the physiology of FC. Increased FC is often conceived as compensatory reallocation or dedifferentiation^52^,^53^,^54^,^55^. Inflammation may modulate the compensatory process^46^. In the early-course of schizophrenia, proinflammatory cytokines (i.e., interleukin-6) activate the astrocytes and show hyperfunction (increased blood flow and metabolism)^56^. Regional hyperfunction can contribute to increased FC and activity of this region. Moreover, increased connectivity in the DMN has been found in early-course patients with schizophrenia^46^. As mentioned before, pathophysiological process may start before the disease onset^51^. Therefore, it is no wonder that the UHR subjects and the patients exhibit increased cerebellar-DMN connectivity in this study.

MPFC and PCC/precuneus are key nodes of the DMN, and act as key players in self-referential processing and emotional regulation^57^,^58^,^59^. Increased cerebellar-DMN connectivity may have an effect on the function of the DMN, and lead to cognitive and emotional disturbances in the UHR subjects and the patients^4^. It can also enhance the risk for the UHR subjects to transit to psychosis. Specially, positive correlations are found between the mean *z* values of the right Crus I-bilateral precuneus connectivity and the SIPS negative symptoms scores/total scores, and the PANSS negative symptoms scores/total scores in the UHR subjects, indicating that the cerebellar-DMN connectivity bears clinical significance. However, these significant correlations disappear in the patients. It is speculated that alterations in cerebellar-DMN connectivity might have occurred around the disease onset in first-episode schizophrenia with predominantly positive and negative symptoms, complicating the correlations between clinical symptoms and abnormal cerebellar-DMN connectivity. Previously, significantly positive correlation was found between the thalamo-orbitofrontal cortex connectivities and Global Assessment of Functioning scores in UHR subjects, but this correlation was not observed in first-episode patients^60^. The previous study gives support to our speculation.

The present study has several limitations in addition to relatively small sample size. First, the present study is cross-sectional, and which part of the UHR subjects will convert to psychosis subsequently remains unclear. Therefore we do not know the difference of the cerebellar-DMN connectivity between the UHR subjects who will convert to psychosis later and those who will not. A follow-up study is needed to elucidate this issue. Second, we used the cerebellar seeds linking to the DMN. This method enhances the specificity of the findings from the DMN. Meanwhile, other connectivity is neglected in this study. Third, the inhomogeneous B0 field induced distortion usually happens at the prefrontal cortex and the cerebellum, where the present study mainly focuses on. Hence, the present findings should be interpreted with caution. Future study should be designed to correct he inhomogeneous B0 field induced distortion. Finally, the MRI data are acquired at resting state with a relatively long repetition time. Physiological noise such as heart and respiratory rhythm may have an effect on the data. More rigorous methods need to be developed to minimize such physiological noise.

In conclusion, our findings indicate that the UHR subjects and the patients exhibit increased cerebellar-DMN connectivity. Increased cerebellar-DMN connectivity shared by the UHR subjects and the patients may be a trait alteration for psychosis. Future studies should include longitudinal, multimodal imaging techniques to specify the possibility for cerebellar-DMN connectivity serving as predictors for transition to psychosis in the UHR subjects.

## Additional Information

**How to cite this article**: Wang, H. *et al.* Patients with first-episode, drug-naive schizophrenia and subjects at ultra-high risk of psychosis shared increased cerebellar-default mode network connectivity at rest. *Sci. Rep.* **6**, 26124; doi: 10.1038/srep26124 (2016).

## Supplementary Material

Supplementary Information

## Figures and Tables

**Table 1:** Characteristics of participants.

	UHR subjects (n = 34)	Patients (n = 31)	Controls (n = 37)	*p*
Sex (male/female)	21/13	19/12	18/19	0.45^a^
Age (years)	21.50 ± 3.53	20.61 ± 4.42	20.76 ± 3.08	0.57^b^
Education level (years)	6.26 ± 4.13	6.26 ± 4.27	5.46 ± 1.87	0.55^b^
FD (mm)	0.10 ± 0.04	0.10 ± 0.05	0.08 ± 0.04	0.37^b^
Illness duration (months)		5.16 ± 3.18		
SIPS				
positive symptoms	9.59 ± 3.10			
negative symptoms	13.18 ± 6.55			
disorganized symptoms	6.41 ± 3.18			
general symptoms	6.38 ± 3.52			
total scores	35.56 ± 11.05			
PANSS				
positive symptoms	13.47 ± 2.49	20.32 ± 4.15	7.11 ± 0.39	<0.001^b^
negative symptoms	16.32 ± 5.49	20.00 ± 6.14	7.05 ± 0.23	<0.001^b^
total scores	66.06 ± 11.51	82.90 ± 16.95	30.78 ± 1.36	<0.001^b^

UHR = ultra-high risk, FD = framewise displacement, SIPS = structured interview for prodromal syndromes, PANSS = Positive and Negative Symptom Scale.

^a^The *p* value for sex distribution was obtained by chi-square test.

^b^The *p* values were obtained by analysis of variance (ANOVA).

**Table 2:** Brain regions with abnormal cerebellar connectivity between groups.

Cluster location	Peak (MNI)			Number of voxels	*T* value
	x	y	z		
*Seed: Left Crus I*					
*UHR subjects > Controls*					
Left middle temporal gyrus	−66	−27	−6	35	3.8290
*UHR subjects < Controls*					
None					
*Patients vs Controls*					
None					
*Patients > UHR subjects*					
Right superior MPFC	6	45	30	26	3.5324
*Patients < UHR subjects*					
None					
*Seed: Right Crus I*					
*UHR subjects > Controls*					
Bilateral precuneus	0	−54	18	22	3.2874
Left cuneus	−15	−63	21	21	3.2021
Bilateral PCC/precuneus	3	−57	30	73	3.3506
*UHR subjects < Controls*					
None					
*Patients > Controls*					
Left middle temporal gyrus	−63	−18	−12	77	4.7358
Bilateral rectus	9	45	−15	30	3.9912
Bilateral PCC/precuneus/cuneus	−9	−45	33	399	4.9180
Right AG	54	−63	27	20	3.3670
*Patients < Controls*					
None					
*Patients vs UHR subjects*					
None					
*Seed: lobule IX*					
*UHR subjects > Controls*					
Bilateral superior MPFC/ACC	12	39	18	224	5.0566
Right middle frontal gyrus	30	24	48	21	4.0687
*UHR subjects < Controls*					
None					
*Patients > Controls*					
Left superior MPFC	−12	45	15	26	4.0016
*Patients < Controls*					
None					
*Patients > UHR subjects*					
None					
*Patients < UHR subjects*					
Bilateral superior MPFC/ACC	3	39	21	84	−3.9892
Right middle frontal gyrus	27	18	45	22	−3.7194

MNI = Montreal Neurological Institute; MPFC = medial prefrontal cortex; PCC = posterior cingulate cortex; AG = angular gyrus; ACC = anterior cingulate cortex.
