# Supplementary material for: Patients with first-episode, drug-naive schizophrenia and subjects at ultra-high risk of psychosis shared increased cerebellar-default mode network connectivity at rest
Source: Sci Rep. 2016 May 18;6:26124. doi: 10.1038/srep26124 (PMC4870637; doi:10.1038/srep26124)
Supplement: Supplementary Information [file srep26124-s1.pdf]

Title:

Patients with first-episode, drug-naive schizophrenia and subjects at ultra-high risk of psychosis shared increased cerebellar-default mode network connectivity at rest

Authors:

Houliang Wang[1] Wenbin Guo[1] Feng Liu[2] Guodong Wang[1] Hailong Lyu[1] Renrong Wu[1] Jindong Chen[1] Shuai Wang[1] Lehua Li[1] Jingping Zhao[1]

Affiliation/address:

1. Mental Health Institute of the Second Xiangya Hospital, Central South University, The China National Clinical Research Center for Mental Health Disorders，National Technology Institute of Psychiatry, Key Laboratory of Psychiatry and Mental Health of Hunan Province, 139 Middle Renmin Road, Changsha, Hunan 410011, People's Republic of China.

2. Key Laboratory for NeuroInformation of Ministry of Education, School of Life Science and Technology, University of Electronic Science and Technology of China, Chengdu, Sichuan, China.

# Corresponding authors:

Wenbin Guo

Mental Health Institute of the Second Xiangya Hospital, Central South University, The China National Clinical Research Center for Mental Health Disorders，National Technology Institute of Psychiatry, Key Laboratory of Psychiatry and Mental Health of Hunan Province, 139 Middle Renmin Road, Changsha, Hunan 410011, People's Republic of China.

E-mail: guowenbin76@163.com

Tel.: +86 731 85360921

Jingping Zhao
Mental Health Institute of the Second Xiangya Hospital, Central South University, The China National Clinical Research Center for Mental Health Disorders，National Technology Institute of Psychiatry, Key Laboratory of Psychiatry and Mental Health of Hunan Province, 139 Middle Renmin Road, Changsha, Hunan 410011, People's Republic of China.
E-mail: zhaojingpingcsu@163.com
Tel.: +86 731 85360921

MRI acquisition and Image preprocessing

MRI scans were acquired on a Siemens 3T scanner. The participants were asked to remain motionless and awake with their eyes closed. Soft earplugs and foam pads were used to reduce scanner noise and head motion. Resting-state functional scans were obtained with a gradient-echo echo-planar imaging (EPI) sequence using the following parameters: repetition time/echo time = 2000 ms/30 ms, 33 slices, 64 × 64 matrix, 90° flip angle, 22 cm field of view, 4 mm slice thickness, no slice gap, and 240 volumes (480 s). High-resolution T1-weighted scans were also obtained with a three-dimensional spoiled gradient-recalled sequence in an axial orientation: repetition time/echo time = 8.1 ms/3.7 ms, 7° flip angle, 25.6 cm field of view, 256 × 256 matrix, 188 slices, 1 mm slice thickness, no slice gap.

Data Processing Assistant for Resting-State fMRI (DPARSF) software [1] was used to preprocess the data. After slice timing and head motion correction, participants with more than 2 mm of maximal translation and 2° of maximal rotation were excluded. To correct the inhomogeneous B0 field induced distortion, we performed the following steps [2] (Figure S5). First, the T1 scans were reoriented and coregistered to the mean functional images. Subsequently, the T1 images were segmented into gray matter, white matter, and cerebrospinal fluid by using a unified segmentation algorithm [3]. Next, the motion-corrected functional volumes were spatially

normalized to the MNI space and resampled to 3 × 3 × 3 mm^3 voxels using the normalization parameters estimated during unified segmentation. After normalization, the images were smoothed (with a 4 mm full width at half maximum Gaussian kernel), bandpass filtered (0.01 - 0.08 Hz), and linearly detrended. Several covariates, including Friston-24 head motion parameters obtained by rigid body correction, signal from a ventricular region of interest (ROI), and signal from a region centered in the white matter, were removed. The global signal was not removed as indicated in a previous study [4].

FC analysis with the software REST

The software REST is a wrapper of existing algorithms and utilities [5]. The steps to perform FC analysis are as follows. First, we press the "Fun. Connectivity" button in Figure S3, and the interface switches to Figure S4. Then, we set the Data Directory, and press the "Voxel wise" button in Figure S4, and set the ROI to perform FC analysis. Finally, we press the "Do all" button, and FC analysis runs automatically. Pearson correlation coefficients between each seed and other voxels of the entire brain were computed to create correlation maps that were *z*-transformed with Fisher's *r*-to-*z* transformation. For more details, please see a previous study [5].

References

1 Yan, C. & Zang, Y. DPARSF: A MATLAB toolbox for "pipeline" data analysis of resting-state fMRI. *Front Syst Neurosci* **4**, 13 (2010).
2 Kybic, J., Thevenaz, P., Nirkko, A. & Unser, M. Unwarping of unidirectionally distorted EPI images. *IEEE Trans Med Imaging* **19**, 80-93 (2000).
3 Ashburner, J. & Friston, K. J. Unified segmentation. *Neuroimage* **26**, 839-851 (2005).
4 Hahamy, A. *et al.* Save the global: global signal connectivity as a tool for studying clinical populations with functional magnetic resonance imaging. *Brain Connect* **4**, 395-403 (2014).
5 Song, X. W. *et al.* REST: a toolkit for resting-state functional magnetic resonance imaging data processing. *PLoS One* **6**, e25031 (2011).

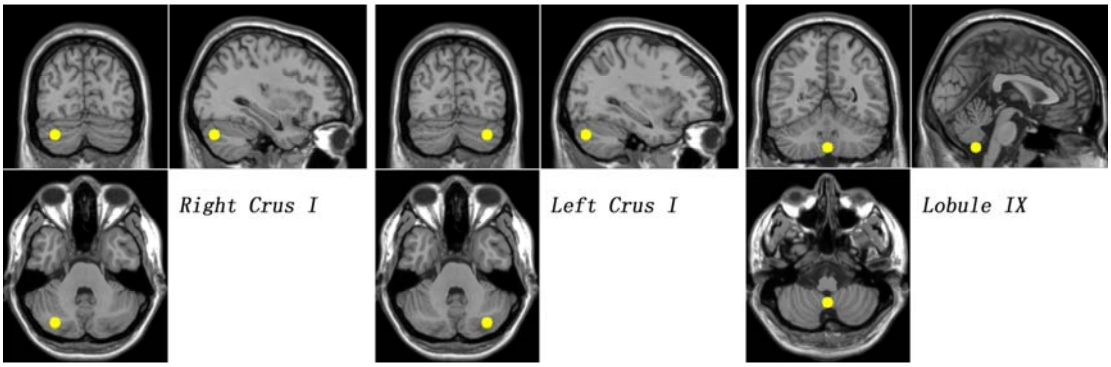

Figure S1. Six mm-radius sphere cerebellar seeds for the seed-based FC analysis. FC = functional connectivity.

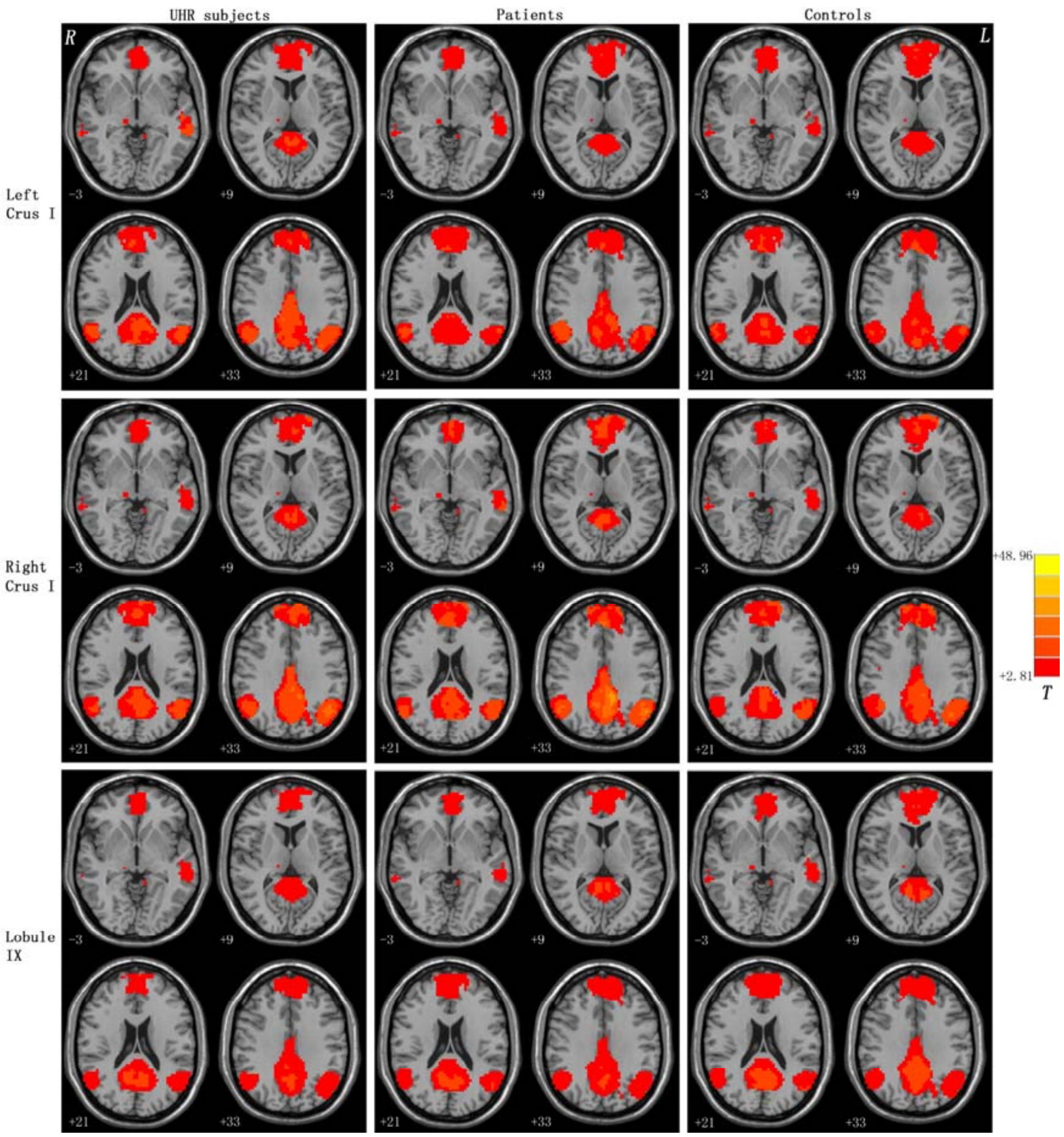

Figure S2. The cerebellar seeds have FCs with the default mode network in the UHR subjects, the patients and the controls. Red denotes increased FC values. Color bar indicates *t* values from one-sample *t*-tests. FC = functional connectivity, UHR = ultra-high risk.

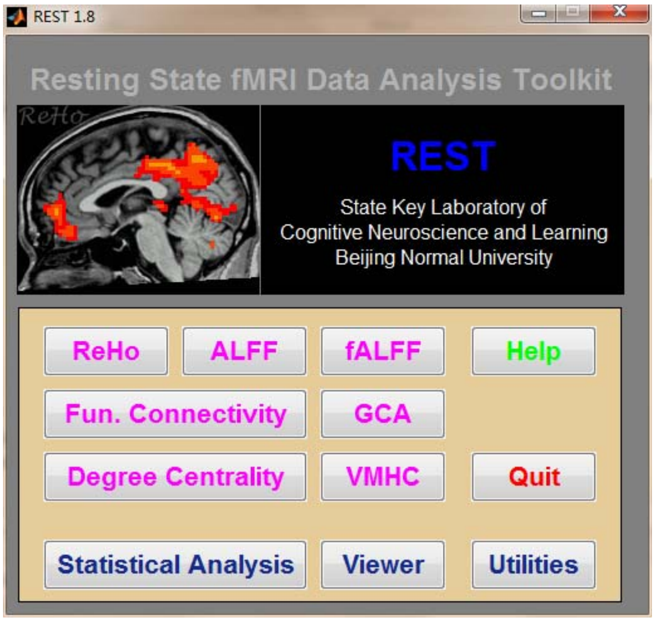

Figure S3. FC analysis with the software REST.

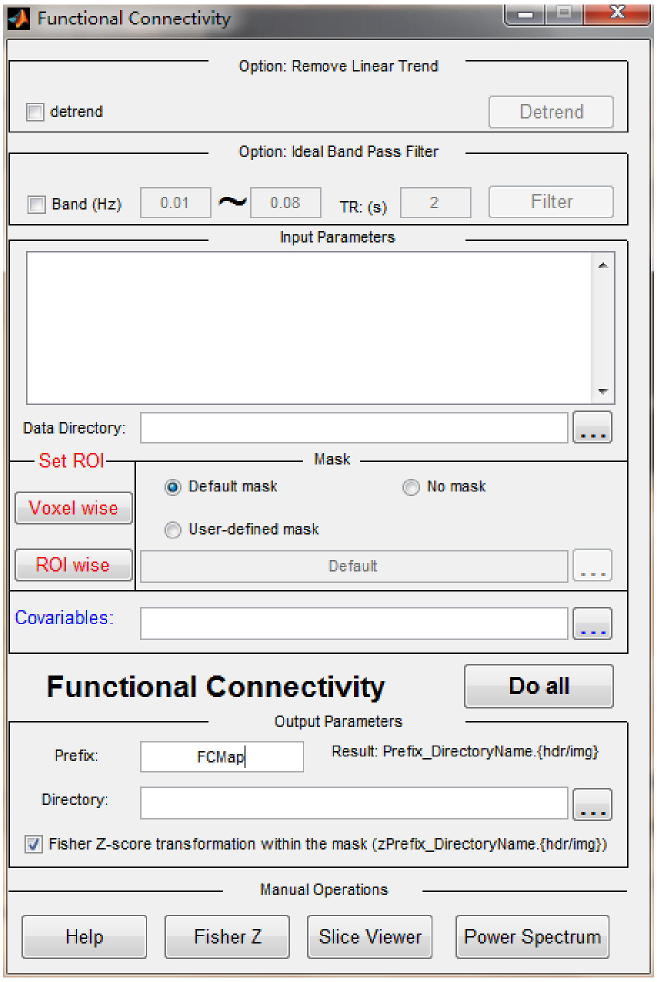

Figure S4. FC analysis with the software REST.

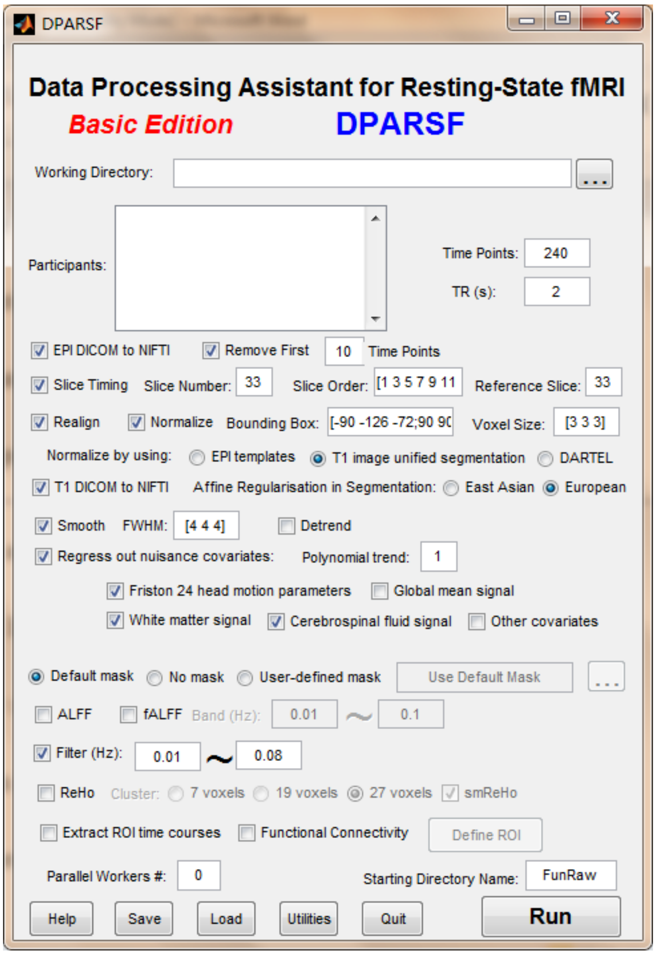

Figure S5. Image preprocessing with the Data Processing Assistant for Resting-State fMRI (DPARSF) software.
